# Collegiate women’s wrestling body fat percentage and minimum wrestling weight values: time for revisiting minimal body fat percent?

**DOI:** 10.1080/15502783.2024.2304561

**Published:** 2024-01-16

**Authors:** Andrew R. Jagim, Grant M. Tinsley, Robert A. Oppliger, Craig A. Horswill, Ward C. Dobbs, Jennifer B. Fields, Cliff Cushard, Paul D. Rademacher, Margaret T. Jones

**Affiliations:** a Sports Medicine, Mayo Clinic Health System, La Crosse, WI, USA; b Department of Exercise and Sport Science, University of Wisconsin – La Crosse, La Crosse, WI, USA; c Department of Kinesiology & Sport Management, Energy Balance & Body Composition Laboratory Texas Tech University, Lubbock, TX, USA; d Department of Exercise Science, University of Iowa, Iowa City, IA, USA; e Kinesiology and Nutrition, University of Illinois Chicago, Chicago, IL, USA; f Department of Nutritional Sciences, University of Connecticut, Storrs, CT, USA; g Athletics, Indiana Institute of Technology, Fort Wayne, USA; h Athletics, Adrian College, Adrian, MI, USA; i Sport, Recreation, and Tourism Management, George Mason University, Fairfax, VA, USA

**Keywords:** Wrestling, body composition, minimal wrestling weight, body fat, weight certification

## Abstract

**Background:**

The estimation of body fat percentage (BF%) in wrestling is used to determine the minimum wrestling weight (MWW) and lowest allowable weight class (MWC) in which wrestlers are eligible to compete. A 12% minimum threshold is currently used for women wrestlers, yet a potential increase for safety has been discussed. Because of the novelty of collegiate women’s wrestling, there is a paucity of literature available on the body composition norms of this population. The purpose of this study was to provide a descriptive summary of BF% and MWW values of female wrestlers and how MWW values would change with the use of different BF% thresholds.

**Methods:**

Data from the 2022–2023 collegiate season were retrospectively analyzed resulting in a sample of 1,683 collegiate women wrestlers from the National Association of Intercollegiate Athletics (NAIA, *n* = 868) and the National Collegiate Athletics Association (NCAA, *n* = 815). All wrestlers completed skinfold assessments for weight certification at the start of the competition season. The skinfold values were used to estimate BF% using the Slaughter skinfold prediction equation. Frequency statistics and descriptive analysis were performed to compute normative MWW and BF% profiles. BF% thresholds of 12% (12MWW) and the BF% value defined as the lowest 5th percentile, which would be considered unusually lean, were used to determine the resulting MWW and MWC for each method. The lowest recorded weight and weight class division throughout the season was also recorded for each wrestler.

**Results:**

There was a positively skewed (0.94) and platykurtic (1.86) distribution of MWW values. The median ± interquartile range BF% for all wrestlers was 27.4 ± 10.22%, with 17% BF representing the 5th percentile. Only 354 out of 1,579 (22.4%) wrestlers competed in their lowest allowable weight class, based on the 12MWW. Of these 354 wrestlers, the mean BF% was 21.3 ± 5.2% at weight certification with only *n* = 17 being at or below 12% body fat and an average weight loss of 11.1 ± 8.8 lbs. from the time of weight certification. Throughout the season, wrestlers competed at weights that were, on average (mean ± SD), 19.4 ± 16.9 lbs. higher than their 12MWW (95% CI: 18.6, 20.2 lbs. *p* < 0.001; effect size [ES] = 1.1), 13.4 ± 19.0 lbs. higher than the 17MWW (*p* < 0.001; ES = 0.70), and 8.7 ± 8.3 lbs. lower than their weight at the certification (95% CI: 8.3, 9.1 lbs. *p* < 0.001; ES = 1.1).

**Conclusions:**

Nearly all BF% values were well above the 12% threshold used to determine MWW. Increasing the minimum BF% threshold from 12% to 17% would affect a small percentage of wrestlers, likely reduce the need for excessive weight cutting, and minimize the deleterious health effects of an athlete at such a low BF%.

## 1. Introduction

Since 2004, when women’s wrestling made its debut as an official sport in the Olympics, participation in wrestling by women has grown immensely within the United States (US) at the collegiate level. As of 2023, approximately 1,944 women athletes were competing in collegiate wrestling [1]. There are currently multiple schools across the US that have club teams or compete as an emerging sport at the Division I, II, or III levels within the National Collegiate Athletic Association (NCAA) as well as other collegiate athletic associations (e.g. National Association of Intercollegiate Athletics or NAIA, National Junior College Athletic Association and California Community College Athletic Association). As such, women’s wrestling is on track to become a sanctioned varsity sport in the NCAA at the Division I level.

As in other collegiate sports, the safety and equal opportunity of women wrestlers are paramount concerns to the NCAA, NAIA, and other sport-governing bodies (SGB). To that end, wrestling requires athletes to compete in designated weight classes for parity in competition and injury reduction. Unfortunately, despite well-established practices for safe and conservative weight loss strategies for athletes, previous research has indicated that wrestlers and combat sport athletes rely on unhealthy practices such as extended fasting periods, dehydration strategies, excessive exercise, and the use of weight loss pills or laxatives at times [2–5]. These practices can pose a significant risk to the health and well-being of the athlete.

As a counter to harmful weight loss practice, SGBs now require wrestlers to complete a weight certification process that consists of a bodyweight assessment, urine specific gravity test (to confirm an euhydrated state), and a body composition test to estimate body fat percentage (BF%) and fat-free mass (FFM) during the pre-season period. These values are then used in the online system provided by the National Wrestling Coaches Association to determine the minimal wrestling weight for each athlete by extrapolating what their weight would be (assuming no changes in FFM) if they were to wrestle at a body fat percentage of 5% (college) or 7% (high school) for males, and 12% for females [1]. These values have been agreed upon by the NCAA, NAIA, National Collegiate Wrestling Association, and state high school athletic associations in an attempt to prevent wrestlers from excessive weight reduction to compete at unreasonably low weight classes and predispose them to hyperthermia (heat stroke), and other health concerns such as Relative Energy Deficiency in Sport (RED-s) and the female athlete triad [6–9].

Recently, there has been concern raised regarding the use of the 12% body fat threshold currently used for MWW determination in women wrestlers [10]. At the time of inception, there was limited available evidence indicating that this was a safe degree of body fatness for young active women to be competing at. Early work [11] has proposed that a BF% of 17% may be needed for menarche with ~20% required for the maintenance of normal menstrual cycle function, which is well above the 12% currently used in wrestling. In clinical populations, BF% values of 23% have been identified as the degree of body fatness required for menstrual recovery in patients with anorexia nervosa [12,13]. However, to date, there are limited data available supporting a specific threshold for BF% in wrestlers that could be established as a safe BF% to compete at for wrestlers, while minimizing the deleterious health and performance effects of low body fatness. To mitigate the potential risks, there has been consideration to raise this threshold to reduce the pressures on young women wrestlers for excessive weight loss and to help prevent risk factors of RED-s, which could lead to the female athlete triad [6–8,14,15]. If adopted, this would change the MWW values of athletes competing, and subsequently, the minimal weight class they would be eligible to compete in, along with the overall distribution of wrestlers in each weight class division. As such, this type of change could require the development of new weight class divisions.

Currently, there are no data available describing the pre-season weight, estimates of BF%, and MWW values for collegiate women wrestlers. Therefore, the purpose of this study was to provide a descriptive summary of BF% and MWW values of female wrestlers and how MWW values would change with the use of different BF% thresholds. A secondary aim was to evaluate whether a newly proposed BF% threshold could be a reasonable minimum threshold for MWW determination.

## 2. Materials and methods

### 2.1. Participants

A total of 1,683 collegiate women wrestlers were included in the analysis. Data were extracted from the weight certification submission system (Optimal Performance Calculator [OPC]) provided by the NWCA [1] and deidentified to maintain the privacy and confidentiality of the wrestlers. No identifiers were included with the data, so age was not available. Consequently, IRB approval was not required, and therefore no informed consent was obtained.

### 2.2. Study design

Data from the 2022–2023 collegiate season were retrospectively analyzed from a sample of 1,683 collegiate women wrestlers competing in the National Association of Intercollegiate Athletics (NAIA, *n* = 868) and the National Collegiate Athletics Association (NCAA, *n* = 815). A cross-sectional design was used to evaluate data obtained at the start of the competition season.

All wrestlers completed skinfold assessments for weight certification at the start of the competition season no sooner than September 1st and by November 1st at the latest [16]. The skinfold thickness was measured by trained testers, typically athletic trainers or others on the sports medicine staff at the local university, and the values were then entered into the OPC system to subsequently compute an estimate of BF% using the Slaughter skinfold prediction equation [17] as required by the NWCA and SGBs [16]. For women, this equation uses two sites, the triceps and subscapular sites. Measurements were taken three times at each site and the average was used for a prediction of BF%. For this study, the pre-season body weight (identified as current weight) and estimated BF% values were extracted for analysis and used to calculate the corresponding MWW, and minimal weight class (MWC) based on the existing and proposed BF% thresholds. The current weight represents an off-season weight before the initiation of wrestling-specific conditioning and weight management. An analysis of percentiles was first conducted to examine what BF% demarcates the leanest 5% of this population and, with rounding to the closest whole percentage, using this value as a proposed threshold for the new minimum BF% for MWW determination. In addition, for each wrestler, the lowest recorded body weight and actual weight class competed in (the lowest weight class) during the season were captured from the NWCA dataset for our analysis.

### 2.3. Minimal wrestling weight determination

MWW was determined by BF% thresholds of 12% (as standard practice), and a newly proposed 17% to determine the resulting MWW and MWC for each method, respectively. The equations below were used for each MWW.

$$12\%MWW=\frac{\left(1-\frac{BF\%}{100}\right)\times Bodyweight}{0.88}$$

$$17\%MWW=\frac{\left(1-\frac{BF\%}{100}\right)\times Bodyweight}{0.83}$$

### 2.4. Statistical analysis

All non-normally distributed data are presented as median ± interquartile range. Frequency statistics and descriptive analysis were completed to compute normative MWW and BF% profiles. Using the percentile results, the body fat at which 5% or less of this population naturally exists was defined as unusually lean and the potential new minimum threshold for BF%. Additionally, the current weight at the time of weight certification and the lowest recorded weight were also used for descriptive purposes and to identify the weight class the wrestler would compete in assuming no weight loss throughout the season or how different the current or lowest recorded body weights compared to each MWW. Normality was assessed via visual inspection of normal Q-Q plots and skewness/kurtosis values. To determine which methods differed from the current weight, a one-way analysis of variance (ANOVA) test with repeated measures was performed, with the MWW determination method specified as a within-subjects factor. Significant effects were followed up with pairwise t-tests and Bonferroni corrections, with the current weight specified as the reference group. All analyses were completed using the Statistical Package for the Social Sciences (v26; SPSS Inc., Chicago, IL). Data are reported using mean differences and 95% confidence intervals where appropriate. Significance was set at *p* < 0.05. Effect sizes (Cohen’s d) were reported, where appropriate, and interpreted as large (d => 0.8), moderate (d = 0.8–0.5), small (d = 0.49–0.20), and trivial (d < 0.2) [18].

## 3. Results

**Figure 1.**
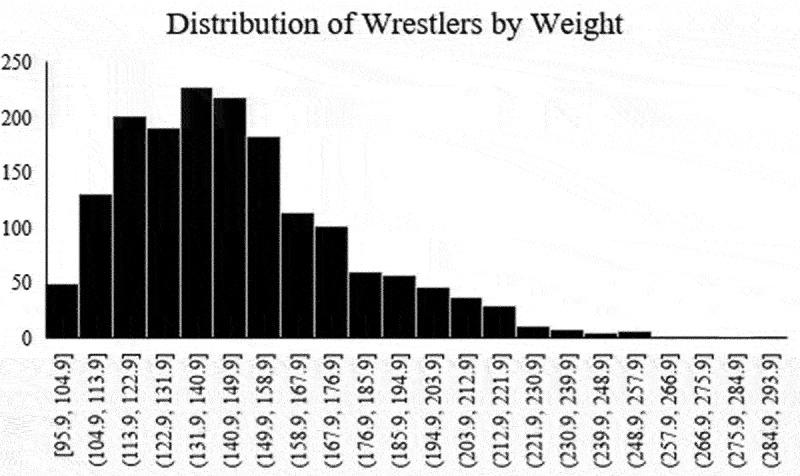
Histogram displaying the distribution of current body weights.

**Figure 2.**
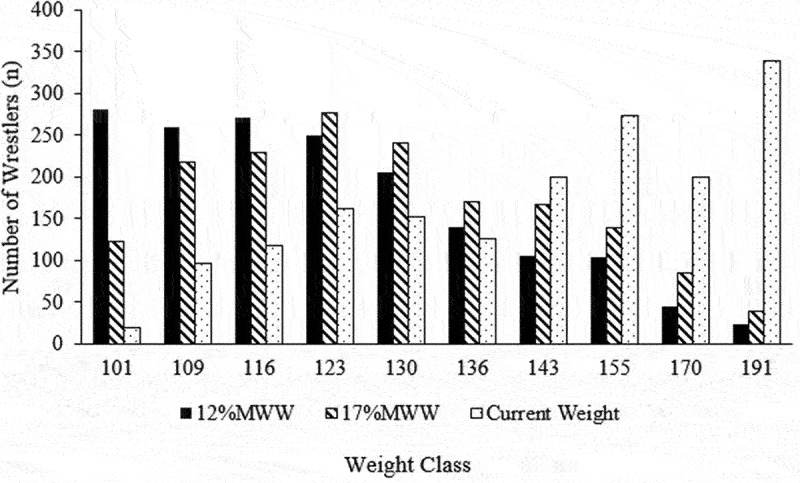
Distribution of wrestlers across each weight class, depending on BF% threshold for MWW and current weight.

There is a positively skewed (0.94) and platykurtic (1.86) distribution of MWW values based on the 12% BF% threshold (Figure 1). The median ± interquartile range BF% for all wrestlers was 27.4 ± 10.2%. The 5th, 25th, 50th, 75th, and 95th percentiles for BF% were 17.2%, 22.6%, 27.4%, 32.9%, and 43.2%, respectively. As such, a value of 17% was used for the newly proposed BF% threshold to determine MWW.

(Figure 2) provides a summary of the distribution of wrestlers in each weight class, depending on the BF% threshold used for MWW determination.

**Figure 3.**
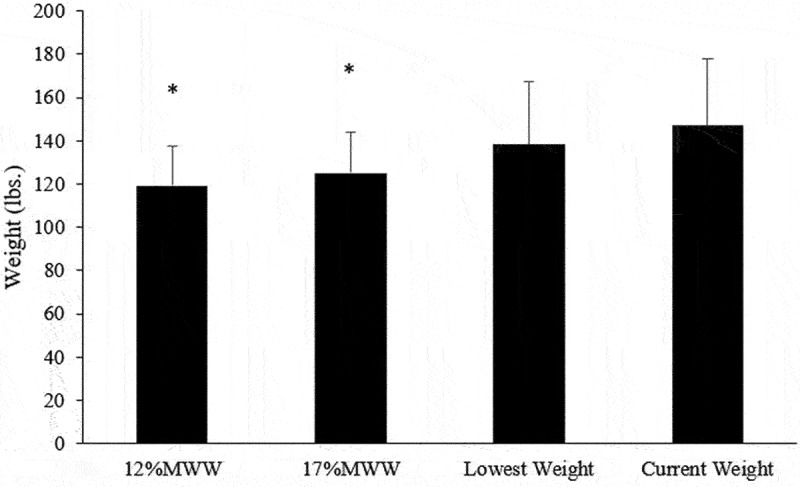
Mean and SD for minimal wrestling weight compared to lowest recorded and current weight. *Denotes significant difference from current weight.

The MWW values calculated using the 12% and 17% BF% thresholds were significantly lower than the lowest recorded weight and the current weights of the wrestlers (Figure 3).

The 12MWW and 17MWW values were (mean difference ± SD; 95% Confidence Intervals, Effect Size) 28.1 ± 17.3 lbs., 95% CI: 27.3, 28.9 (*p* < 0.001; ES = 1.6) and 22.1 ± 19.2 lbs., 95% CI: 21.2, 23.0 (*p* < 0.001; ES = 1.1) lower than the current weight, respectively, as seen in Table 1. Only 354 out of 1,579 (22.4%) wrestlers competed in their lowest allowable weight class, based on the 12MWW. Of these 354 wrestlers, the mean BF% was 21.2 ± 5.2%, and *n* = 17 wrestlers were at or below 12% BF at the time of weight certification. Eight wrestlers were at their 12MWW at the time of weight-certification.

**Table 1.** Current weight and minimal wrestling weight as determined by each BF% threshold.

	MWW	5th	25th	50th	75th	95th
12%	119.2 ± 18.6*^≠	93.6	105.9	116.7	129.8	153.1
17%	125.2 ± 18.8*#≠	98.7	111.8	122.9	136.3	159.6
Lowest Weight	138.4 ± 29.2*^#	100.2	116.0	134.2	153.4	190.0
Current Weight	147.2 ± 30.6^#≠	107.2	124.2	142.0	163.0	207.0

Data presented as means ± standard deviation. *Denotes statistical significance at *p* < 0.05. MWW = minimum wrestling weight; lbs. = pounds. *Denotes significant difference from current weight; #Denotes significant difference from 12%^Denotes significant difference from 17%; ≠ Denotes significant difference from the lowest recorded weight.

The lowest recorded weight for all wrestlers registered throughout the season was 19.4 ± 16.9, 95% CI: 18.6, 20.2 lbs. (*p* < 0.001; ES = 1.1) higher than their 12MWW, and 8.7 ± 8.3, 95% CI: 8.3, 9.1 lbs. (*p* < 0.001; ES = 1.1) lower than their current weight at the time of weight certification. The lowest recorded weight was 13.4 ± 19.0 lbs. (*p* < 0.001; ES = 0.70) higher than the 17MWW.

**Figure 4.**
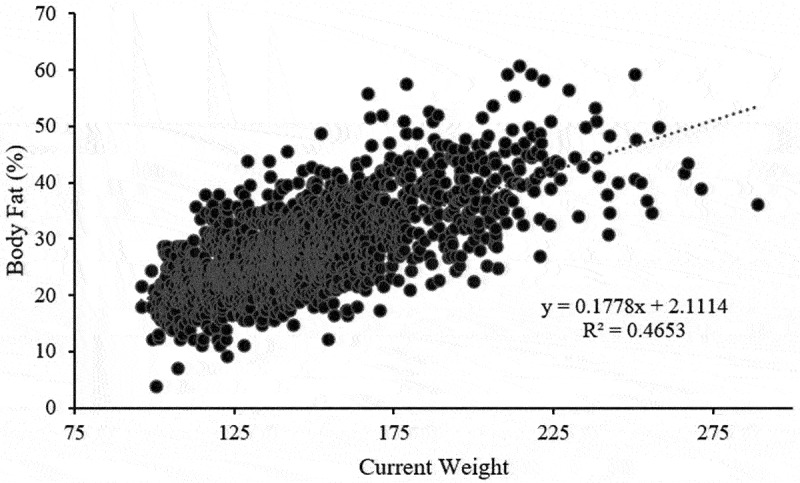
Linear regression of body fat percentage and current body weight.

Linear regression indicated that body weight was a significant predictor of BF% (R^2^ = 0.47; β = 0.686; SEE = 5.8), accounting for 47% of the variance in BF% across all wrestlers as seen in Figure 4.

## 4. Discussion

The primary aim of the current study was to provide a descriptive summary of BF% and MWW values of collegiate women wrestlers. The median BF% was 27.4% with findings from the current study indicating that 95% of the female wrestlers from the 2022–2023 pre-season were above 17% body fat. This is a noteworthy observation, as when traditional probability is applied [19], we conclude that it is a rare event to find women wrestlers below 17% body fat when using the current standard practices for BF% determination. Yet 12% body fat is currently used as the threshold for determining the minimal wrestling weight class in women [20]. The 12% value was initially set using theoretical models, limited cadaver analyses, and the assumption that 12% would provide an adequate mass of stored energy and lipid precursors to sustain good health and performance in female athletes [21]. The current observation is persuasive when considering an increase from the 12% to 17% BF% threshold, which would be more reasonable given that the pre-season median BF% was 27.4%. Large effect sizes were observed regarding the average magnitude of weight difference between the wrestlers’ current weight and the resulting MWW based on the BF% threshold used. When using the 12% BF% threshold currently used for MWW determination in collegiate wrestling, on average wrestlers were 28.1 ± 17.3 lbs. (ES = 1.6) above their eligible MWW at the time of weight certification. Only 354 out of 1,579 (22.4%) wrestlers competed in their lowest allowable weight class, based on the 12MWW. Of the 354 wrestlers that competed in their lowest allowable weight class, their mean BF% (~21%) was still well above a 12% threshold (at the time of weight certification). On average, of these 354 wrestlers, the average weight loss was approximately 11 lbs. from the time of weight certification to their lowest recorded weight in competition. Even more noteworthy is that the lowest weight that all wrestlers achieved throughout the season was 19 lbs. higher than their 12MWW, and 9 lbs. lower than their weight at the time of weight certification. It is important to note that the lowest weight values do not include a measurement of hydration status, so it is unknown how much of the reported weight loss was attributed to rapid weight cutting practices (e.g. dehydration) rather than reductions in body fat.

A secondary aim was to evaluate how MWW values would change with the adoption of a higher BF% threshold. If a threshold of 17% were to be adopted, wrestlers would still be 22.1 ± 19.2 lbs. (ES = 1.1) above their MWW on average at the time of weight certification, based on 2022–2023 data. The standard recommendation for maximizing fat reduction and minimizing loss of FFM is 0.5 to 1 kg mass loss per week [22]. Presumably, this minimizes the risk of the female athlete triad. Using these guidelines, it would take an athlete nearly 3.5 to 7 months to achieve 12% body fat. If 17% is applied for MWW, the duration for weight loss declines to 2.5 to 5 months. The US college women’s wrestling season spans approximately 4 months [23]. Therefore, whether applying an absolute minimum BF% that is rare or using a safe rate of weight loss that is nearly impossible within the confines of the season, the threshold of 12% body fat seems unreasonable, if not unsafe, as the minimum for women wrestlers. Collectively, these data suggest that raising the BF% threshold from 12% to 17% would impact only a small percentage of wrestlers and would still allow for weight reduction with an emphasis on reducing excess body fat that would be expected to occur with training during the season and allow teams to fill all weight classes while mitigating health risks of the pursuit of low body fat in the wrestlers. We acknowledge that the athletes in this study were assessed in the pre-season and the cutoff for the 5th percentile was generated from women who may not have attempted weight reduction to their leanest potential. However, the concern here is beyond an absolute BF%. Research subsequent to the 1996 ACSM position stand shows the process of creating a negative energy balance by restricting energy intake and/or increasing in energy expenditure to reduce body weight may predispose athletes to RED-s and lead to the harmful effects of the female athlete triad [8]. Importantly, an absolute low BF% does not seem to be required to depress estrogen levels, promote bone mineral loss, and bring about other adverse outcomes [24], rather extended time spent in a low energy state (<30 kcal/kg of FFM) may be the primary causative factor. However, it may be difficult to completely isolate the two as a low state of energy availability would likely lead to reductions in body fat. Regardless, efforts should be made to minimize risks associated with both intentional energy restriction and low body fatness to avoid the negative health consequences associated with both in women athletes.

Based on the body weights at the time of weight certification, there was an unequal distribution of wrestlers, resulting in a positively skewed distribution. As such, the collegiate athletic associations may want to alter weight class divisions accordingly, to best reflect the natural distribution of wrestlers as determined by their weight and MWW. As seen in Figure 2, if a higher BF% threshold is adopted for MWW determination, a higher percentage of wrestlers would be ineligible to compete in the lighter weight class divisions (e.g. 101 and 109) as the resulting MWW would be higher if athletes were not allowed to induce weight loss eliciting a BF% of 12%. Similarly, more athletes would be allocated to higher-weight class divisions, which again alters the distribution of wrestlers across the weight divisions. If such a change were to occur, new weight class divisions may not be needed.

Body weight was a significant predictor of BF%, accounting for 47% of the variance, and for every 1 lbs. increase in weight, there was a corresponding 0.69% increase in BF%. Heavier wrestlers are therefore more likely to have a higher amount of body fat, relative to total body weight, which may allow for more weight loss throughout the season, and subsequently allow for moving down weight class divisions. At the same weight, a higher BF% would result in a lower MWW for two athletes at the same weight and ultimately allow the athlete with a higher BF% to move down to a lower weight class division, depending on the specific MWW. For example, if two athletes weigh 120 lbs., but Athlete A was estimated to be at 32% body fat and Athlete B was estimated to be at 25% body fat, their resulting MWW values would be 93 and 102 lbs., respectively, if using a 12% threshold for BF%. This would allow Athlete A to compete in the 101-weight class division, whereas Athlete B would compete in the 109-weight class division.

Several limitations exist for the current study. The reliance on estimates of BF% that were derived from the Slaughter skinfold prediction equation [17], was recently found to underestimate measures of FFM in female athletes, and therefore overestimate BF%. Specifically, estimates of FFM derived from the Slaughter skinfold prediction equation [17] resulted in a mean difference (SD) of −2.1 kg (3.0) kg, a SEE of 2.1 kg (*p* < 0.05), and a concordance correlation coefficient value of 0.60 when compared to criterion measures that account for variability in the FFM, (i.e. body water), which vary with maturation and possibly type of physical training [25–28]. Further, investigators found a proportional bias towards a greater underestimation of FFM for athletes with higher FFM values, which could subsequently result in a higher estimate of BF% and lower MWW when using the Slaughter equation [17,29]. Theoretically, this could allow a wrestler to compete in a lower weight class than what would be allowed if FFM was assessed more accurately. When investigators [29] evaluated the number of women athletes that would be miscategorized when determining MWW, the use of the Slaughter equation [17] would have allowed 31/51 (60.8%) of the female athletes included in the study to compete in a weight class that would be different from the criterion-derived MWW and resultant weight class. Therefore, proper body composition management is a vital aspect of the sport. Additionally, this highlights the importance of accurate body composition assessment as the use of valid assessment techniques plays a pivotal role in MWW determination, particularly if one method exhibits proportional bias or consistently over/underestimates BF%. Since the current database was dependent on the estimates of BF% derived from the Slaughter equation, the current analysis may not provide the most accurate measure of mean BF% values and appropriately determined MWW. However, this is the approach currently utilized by the NWCA and therefore still provides a valuable summary of values that are used in practice. Moreover, the error of overestimated BF% and the amount of weight a women wrestler could lose would be diminished by raising the percentage of body fat for MWW to 17%. Lastly, a threshold of 17% is more in alignment with previous research indicating a level of body fatness likely required for regular menstrual function [11–13]. If a wrestler were to be measured at a body fat percentage less than 17%, they would still be able to wrestle at the minimal weight class that would correspond to the 17% threshold [16].

In addition, we depended on skinfold data that were measured by sports medicine staff members who differed at each institution and that would have varied in training and experience. Intertester reliability and reliability within the same tester with inexperience adds to the variability of the data [30,31]. The MWW program has been in place for several years, so we assume the assessments are made by seasoned testers. Nonetheless, we cannot confirm the degree of error these factors produced in the current data set. We would expect the errors between teams would not be systemic, and within a team, would be random except for bias due to wrestler size. Presumably, these errors are dampened by the large data set, the largest to date on women wrestlers, but confirmation is needed from future investigations.

## 5. Conclusions

Nearly all (>95%) BF% values were above the 12% threshold currently used to determine MWW by the NWCA. Additionally, very few wrestlers competed in their lowest allowable weight class division throughout the season. Similarly, wrestlers rarely reached the MWW determined using the 12% threshold. Therefore, increasing the minimum BF% threshold from 12% to 17% (5th percentile) would likely only affect a small percentage of wrestlers, likely reduce the need for excessive weight loss, and minimize the deleterious health effects of an athlete competing at a low BF%. Switching to a higher BF% threshold subsequently produces higher MWW values and will preclude certain wrestlers from competing in lower-weight class divisions.
